# Bluish Discolouration of Urine Drainage Tube and Bag in a Female Patient with Spina Bifida, Paraplegia, and Suprapubic Cystostomy

**DOI:** 10.1100/tsw.2007.165

**Published:** 2007-07-03

**Authors:** Subramanian Vaidyanathan, Bakul M. Soni

**Affiliations:** Regional Spinal Injuries Centre, District General Hospital, Southport, PR8 6PN, UK

**Keywords:** purple urine bag syndrome, spina bifida, paraplegia, urine infection

## Abstract

We present a female patient with spina bifida, paraplegia, suprapubic cystostomy, and chronic constipation, who became anxious when she noticed a bluish discolouration of her urine drainage system. Urine microbiology revealed growth of *Providencia stuartii* and *Staphylococcus aureus*. There were no systemic features of infection and, therefore, antibiotics were not prescribed for asymptomatic bacteriuria. This patient was advised to change the urine bag every day, and was prescribed senna to facilitate bowel evacuation. She was reassured that bluish discolouration of the urine drainage tube and bag was a transient, benign phenomenon and not indicative of any underlying pathology. Over the next 7 days, the bluish discolouration gradually faded away. Clinical characteristics of patients who are likely to develop this phenomenon and the underlying biochemical mechanism for bluish discolouration of the urine drainage system are discussed in brief.

## CLINICAL IMAGES

A 40-year-old female, who was born with spina bifida, had undergone continent vesicostomy with Benchekroun hydraulic valve in 1993. This patient developed complete obstruction of the stoma and, therefore, suprapubic cystostomy was performed in 2000. She developed vesical calculi and required suprapubic cystolithotomy in 2001. There was recurrence of vesical calculi in 2005 and electrohydraulic lithotripsy was performed through suprapubic cystostomy. Ultrasound examination of kidneys revealed no calculi, no scarring, and no hydronephrosis.

Biochemical analysis of 24-h urine for calcium, phosphate, and urate revealed no abnormality. Blood test showed slightly low calcium level (2.12 mmol/l; reference range: 2.20–2.60; adjusted calcium: 2.14 mmol/l). Phosphate concentration was normal (0.99 mmol/l; reference range: 0.80–1.50). Crystallographic evaluation of vesical calculus was not performed.

The suprapubic silicone Foley catheter was being changed every 8 weeks and the patient had been replacing urine bag thrice a week. She suffered from chronic constipation. In April 2007, this patient became concerned when she noticed bluish discolouration of the drainage tube and to a lesser degree, the urine bag as well (Fig. 1). However, the urine was a normal yellow colour. The patient had been taking domperidone (10 mg, three times a day), levocitrizine (5 mg, one a day), and omeprazole (20 mg, one daily). Microbiology of the urine showed growth of *Providencia stuartii* and *Staphylococcus aureus*; both organisms were sensitive to gentamicin. Since the patient did not have systemic features of infection, e.g., temperature, feeling unwell, antibiotics were not prescribed. She was advised to change the urine bag every day. For constipation, she was prescribed senna syrup 15 ml daily. Over a period of 7 days, the bluish discolouration of the urine drainage system faded away.

## COMMENT

Purple urine-bag syndrome (PUBS) is a rare phenomenon in which the urine bags turn purple or blue. The condition often causes distress to patients and caregivers. Such discolouration of the urine bag is said to be associated with the female gender, alkaline urine, constipation, institutionalization, the use of a plastic (PVC) urinary catheter, and certain bacteria, such as *Providencia* sp., *Escherichia coli*, *Proteus mirabilis*, and *Klebsiella pneumoniae*[1].

In a case control study of purple urine bags in geriatric wards, a total of 66 bacterial strains, belonging to 12 separate species, were isolated from the urine accumulated in bags, but no causative relationship between bacterial species and PUBS was observed. However, bacteriological studies using fresh urine collected through the catheter showed that the bacterial counts were significantly higher, by 1 to 2 logs, in most samples from patients with PUBS than those from the control group ($p$ = 0.012). These data suggested that a higher bacterial yield in urine acted as the most important factor in PUBS, in combination with other facilitating factors, such as female gender and alkaline urine[2].

Very rarely, drugs (such as amitriptyline, triamterene, methylene blue, and methocarbamol) may cause blue or blue-green discolouration of urine[3]. This patient was not taking any of these drugs. The biochemical reaction responsible for the PUBS begins with tryptophan from the food chain. Bacterial degradation of tryptophan in the gastrointestinal tract leads to excessive production of indole, which is absorbed into portal circulation and converted into indoxyl sulphate in the liver, after a series of detoxification transformations. Indoxyl sulphate is excreted in the urine and is digested into indoxyl by the enzyme sulphatase/phosphatase produced by certain bacteria such as *Pseudomonas aeruginosa*, *Proteus mirabilis*, *Morganella morganii*, *E. coli*, and so on. Indoxyl then changes in alkaline urine into indigo, which is blue, and indirubin, which is red. The intensity of discolouration is variable; the longer the urinary catheter drainage system remains unchanged, the more intense will be the discolouration[4].

## CONCLUSION

PUBS is a rare phenomenon in which the urine bags turn purple or blue. Although discolouration of the urine drainage system may cause distress to patients, physicians should reassure patients and their caregivers that a bluish discolouration of the urinary drainage system is a transient, benign phenomenon and is not indicative of underlying pathology. Asymptomatic bacteriuria in patients with indwelling catheters does not require antibiotic therapy and, therefore, these patients do not require any prescription for antibacterials. Improved hygienic measures, treatment of constipation, and more frequent changes of the urine drainage system should suffice, as indeed shown by the case reported herein.

## ACKNOWLEDGMENT

The authors are grateful to Potter Rees, Serious Injury Solicitors, 14-32, Hewitt Street, Manchester M15 4 GB, United Kingdom for payment of open access fee, which enabled publication of this article under open access so that spinal cord injury patients, carers and health professionals can access this article.
